# Revealing the secrets of malaria parasite interaction with blood group A sugars

**DOI:** 10.1179/2047772413Z.000000000113

**Published:** 2013-03

**Authors:** J Alexandra Rowe

**Affiliations:** Institute of Immunology and Infection Research, University of Edinburgh, UK

Invited commentary on ‘Structural Basis for the ABO Blood-Group Dependence of Plasmodium falciparum Rosetting’, Vigan-Womas et al., PLoS Pathogens’ 2012.

ABO blood group is known to influence an individual’s risk of developing severe malaria.^1^ The precise molecular details underlying this intriguing aspect of malaria host-parasite interaction were unclear. However, a recent paper by Vigan-Womas *et al*^2^ sheds light on the topic by providing a detailed understanding of how malaria parasites interact with ABO blood group sugars on human red cells in rosetting, a process contributing to malaria disease and pathology.

Individuals with blood group O are significantly protected against the life-threatening complications of malaria, compared to those with A or B blood groups.^1^ The life-saving effect of group O is thought to occur due to the impaired ability of *Plasmodium falciparum* parasites to form rosettes - clusters of infected red blood cells binding to uninfected red blood cells - in group O blood.^1^ Rosetting occurs due to specific members of the *P. falciparum* Erythrocyte Membrane Protein one (PfEMP1) family of variant antigens, on the surface of infected red cells, binding to receptors, including the A and B blood group trisaccharides, on uninfected red cells (reviewed in^3^). Rosetting contributes to microvascular obstruction in severe malaria,^4^ leading to hypoxia, acidosis, organ dysfunction and death.

Although it has been known for two decades that human red cell ABO blood group affects the ability of malaria parasites to form rosettes,^5^ the precise details of the interaction remained obscure. Vigan-Womas *et al* now provide the molecular information to explain how rosetting malaria parasites bind to the blood group A sugars. The team used a sophisticated mix of *in vitro* functional studies and insights from crystallography to define the binding site for interaction between the malaria parasite rosetting ligand PfEMP1 and the group A-trisaccharide (GalNAc-α1,3(Fuc-α1,2)Gal). They expressed the extracellular domains from a rosette-mediating PfEMP1 variant (Palo Alto varO) as recombinant proteins, and found that the N-terminal head-structure region (known as NTS-DBLα-CIDR), bound the group A-trisaccharide. They crystallized the PfEMP1 N-terminal region to analyze its structure, and used computer docking to identify potential binding sites for the A-trisaccharide. Site-directed mutagenesis was used to test the effect of specific amino acid substitutions, and key PfEMP1 residues essential for binding were confirmed. The binding site is distinct from the previously identified heparin-binding site in the same region.^6^ The authors suggest that the binding site on PfEMP1 for the blood group A-trisaccharide is conserved in other rosetting PfEMP1 variants, however, further work will be required to validate this claim.

Overall, this work gives new insights into the molecular mechanisms of rosetting, and also provides the first crystal structure of a complete PfEMP1 head-structure, a component found in almost all PfEMP1 variants. Hence, the results have implications for understanding PfEMP1 structure-function relationships more widely. Whether the results can be translated into useful rosette-disrupting therapeutic interventions against severe malaria remains to be seen, but the work of Vigan-Womas *et al* is a valuable contribution towards this goal.
